# Thyroxine Differentially Modulates the Peripheral Clock: Lessons from the Human Hair Follicle

**DOI:** 10.1371/journal.pone.0121878

**Published:** 2015-03-30

**Authors:** Jonathan A. Hardman, Iain S. Haslam, Nilofer Farjo, Bessam Farjo, Ralf Paus

**Affiliations:** 1 The Dermatology Centre, Institute of Inflammation and Repair, University of Manchester, Manchester, United Kingdom; 2 Doctoral Training Centre in Integrative Systems Biology, Manchester Interdisciplinary Bio centre, University of Manchester, Manchester, United Kingdom; 3 The Farjo Hair Institute, Manchester, United Kingdom; 4 Department of Dermatology, University of Muenster, Muenster, Germany; University of Texas Southwestern Medical Center, UNITED STATES

## Abstract

The human hair follicle (HF) exhibits peripheral clock activity, with knock-down of clock genes (*BMAL1* and *PER1*) prolonging active hair growth (anagen) and increasing pigmentation. Similarly, thyroid hormones prolong anagen and stimulate pigmentation in cultured human HFs. In addition they are recognized as key regulators of the central clock that controls circadian rhythmicity. Therefore, we asked whether thyroxine (T4) also influences peripheral clock activity in the human HF. Over 24 hours we found a significant reduction in protein levels of BMAL1 and PER1, with their transcript levels also decreasing significantly. Furthermore, while all clock genes maintained their rhythmicity in both the control and T4 treated HFs, there was a significant reduction in the amplitude of *BMAL1* and *PER1* in T4 (100 nM) treated HFs. Accompanying this, cell-cycle progression marker *Cyclin D1* was also assessed appearing to show an induced circadian rhythmicity by T4 however, this was not significant. Contrary to short term cultures, after 6 days, transcript and/or protein levels of all core clock genes (BMAL1, PER1, clock, CRY1, CRY2) were up-regulated in T4 treated HFs. *BMAL1* and *PER1* mRNA was also up-regulated in the HF bulge, the location of HF epithelial stem cells. Together this provides the first direct evidence that T4 modulates the expression of the peripheral molecular clock. Thus, patients with thyroid dysfunction may also show a disordered peripheral clock, which raises the possibility that short term, pulsatile treatment with T4 might permit one to modulate circadian activity in peripheral tissues as a target to treat clock-related disease.

## Introduction

There is an increasing appreciation for the role of the biological clock and its molecular components in maintaining tissue homeostasis [1–5]. It is now understood that most peripheral tissues exhibit functional, oscillating molecular clock activity which is synchronised by a central master regulator, the suprachiasmatic nucleus (SCN) of the hypothalamus [6–8]. When normal molecular clock activity is altered, e.g. during nightshift work, psycho-emotional stress or through poor diet, normal tissue homeostasis is disrupted triggering or aggravating disease, including metabolic syndrome, Alzheimer’s disease, hypertension, diabetes, and cancer [9–14]. Moreover, clock knock-out mice have an increased number of age-related pathologies, including reduced bone density and life span [15–17]. Finally, it is well-established that the pharmacological effects of drug administration on peripheral tissue functions is greatly dependent on the circadian timing of drug administration [18–20]. Therefore a greater understanding of molecular clock regulation may pave the way for the development of novel therapies aimed at correcting clock dysfunction and maintaining normal tissue function.

Due to the complexity of circadian biology, research has primarily utilised *in vitro* cell culture models [21–23], which cannot capture the complex interactions between difference cell types found within tissues, or animal models [1,5,24–26]. Moreover, it is insufficiently understood how intrinsic oscillatory behaviours found in peripheral human tissues, separate from the SCN, are regulated. Thus, it remains a major challenge for translational chronobiological research to identify clinically relevant, SCN-independent regulators of the human peripheral clock.

The human hair follicle (HF) is an ideal model system for biological research in areas ranging from molecular biology and stem cell biology to systems biology and chronobiology [26–28]. The HF is a skin appendage which undergoes life-long cyclic transformations from an active growth phase (anagen) to a destructive phase (catagen) and a phase of relative quiescence (telogen) [29,30]. The molecular clock is now appreciated in hair cycle control both in mice, where clock activity is highly compartmentalised in anagen HFs [5], with mice lacking the core clock protein *Bmal1* having a delayed onset of anagen [25], and humans, where clock genes/proteins expression has been shown in both human skin and plucked human hair shafts [31].

Yet, a functional role for the peripheral molecular clock in human HF physiology has only recently been identified: *ex vivo*, human HFs not only maintain circadian rhythmicity of core clock gene (*CLOCK*, *BMAL1* and *PER1*) transcription in organ culture, but PER1 protein expression is also highly hair cycle dependent, increasing as HFs enter catagen [26]. Functionally, knockdown of *PER1* or *BMAL1* prolongs HF anagen, implicating PER1, BMAL1 and clock target genes in the regulation of anagen-catagen switching during the human HF cycle [26]. In addition to this, knock-down of either *PER1* or *BMAL1* stimulated human HF pigmentation in a hair cycle-independent manner suggesting the human HF has an intrinsic molecular clock which is indispensable for normal HF activity [32]. For this reason, human HF organ culture is a tractable and clinically relevant research model for understanding how the peripheral clock is regulated.

In the current study, we have examined the role of the thyroid hormone (TH), thyroxine (T4), a frequently administered hormones in clinical medicine, as a regulator of the peripheral clock [33]. THs were selected as, on the one hand, HFs are known to be modulated by THs via signalling through the TH receptor beta [34–36], which prolongs anagen, increases pigmentation, stimulates matrix proliferation and inhibits apoptosis in the human HF. Furthermore, T4 up-regulates the stem cell marker keratin 15 *in situ* after short-term application [34,35], increases mitochondrial activity and biogenesis and transcription of the clock gene, *BMAL1* [37], mimicking some of the some of the effects of clock knock-down in human HFs [26].

On the other hand, THs are known to influence the central clock [11,38,39]. For example, the thyroid gland influences clock circadian oscillations [24] and is essential for seasonal rhythms and mating season timing in mammals and shows diurnal expression patterns [40,41]. Moreover, thyroidectomy alters circadian activity and blunts daily oscillations of PER2 [42] and T4 has been implicated in regulating metabolism [40], a process regulated by the molecular clock [43–45]. However, direct evidence that T4 modulates the peripheral clock in human tissues *in situ* is still missing.

Thus, T4 is a potential clinically relevant candidate for regulating peripheral clock activity, namely in the human HF. As human HF organ culture is an ideally suited, easily accessible model system, in addition to having robust and functional peripheral clock activity [26], we will investigate whether T4 modulates peripheral clock activity in the human system in this physiologically and clinically relevant human mini-organ.

## Results

### Thyroxine is a gender-independent modulator of human HF cycling

First, we asked whether the previously reported anagen-prolonging effect of T4 on micro-dissected, organ-cultured human scalp HFs is robust and gender-independent, [34] using occipital scalp HFs from male patients instead of female fronto-temporal HFs used in the van Beek *et al*. (2008) study. This showed that T4 administration over 6 days resulted in a significantly higher percentage of HFs in anagen (38% [51/129]) compared to the vehicle control (24% [33/133]) (p = 0.0077) (**S1A Fig**). Conversely, in the control group a higher percentage of HFs tended to be in early catagen (48% [64/33]) compared to treated HFs (29% [39/129]) (p = 0.0031) (**S1A Fig)**. This demonstrates that T4 is a potent, gender-independent modulator of human HF cycling *in vitro*, thus confirming T4 as a robust modulator of human HF cycling.

### Intrafollicular clock gene and protein expression is significantly reduced by thyroxine treatment

**Fig 1:**
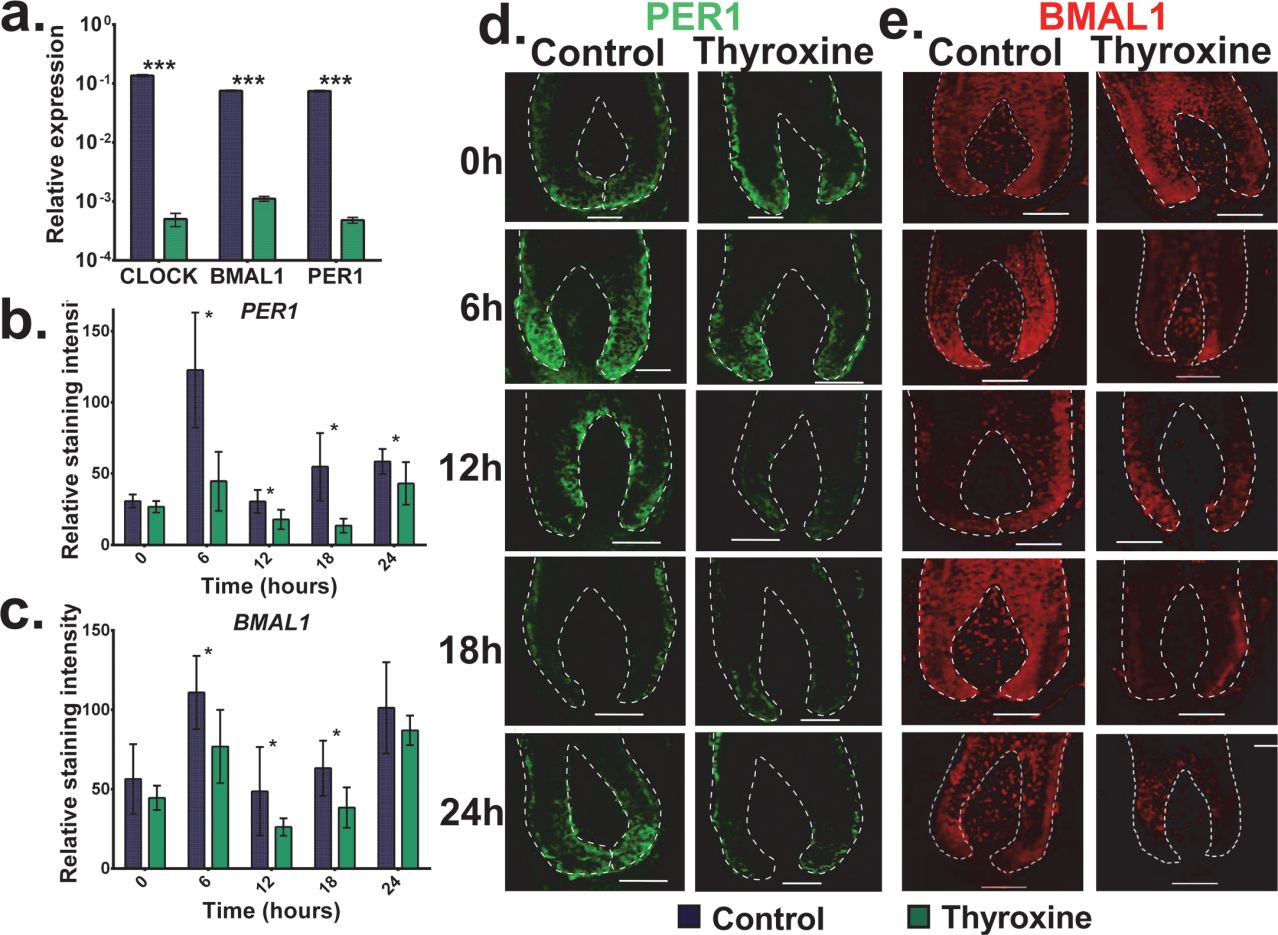
BMAL1 and PER1 protein and transcript levels are reduced by thyroxine treatment. HFs treated with T4 for 24 hours were stained for either BMAL1 or PER1 and transcript levels were assessed. (a) qRT-PCR demonstrated that CLOCK, BMAL1 and PER1, were significantly down regulated by T4 expression (24 hours). Protein expression was also assessed and quantified using immunohistomorphometry. (b,d) PER1 protein was significantly decreased by T4 at 6, 12,18 and 24 hours. BMAL1 protein levels were also decreased significantly by T4 at time points 6,12 and 18 hours showing a tendency to decrease at 0 and 24 hours. (mean (SD) * p < 0.05, *** p < 0.001, Student’s Ttest mean, results were pooled from multiple HFs from 3 patients). (scale bar = 50μm)

Next, we investigated whether T4 treatment impacts on intrafollicular clock gene expression. Quantitative-RT-PCR of HFs treated with T4 for 24 hours demonstrated that there was a significant reduction in gene mRNA steady-state levels for the core clock genes [10,46], i.e. *CLOCK*, *BMAL1* and *PER1* (p< 0.001) (**Fig 1A**). To compliment this we evaluated whether the protein levels were similarly reduced. This was assessed at five time points (0, 6, 12, 18 and 24 hours) to eliminate any effects caused by a shift in circadian rhythmicity. Immunohistomorphometry demonstrated that at 6, 12, 18 and 24 hours there was a significant reduction in PER1 protein levels (p < 0.011), with BMAL1 showing significant reductions at 6, 12, 18 (p<0.016) when compared to vehicle controls (**Fig 1B–1E**). Together these data show that intrafollicular clock gene and protein expression in human anagen scalp HFs is significantly reduced by T4 treatment.

### Thyroxine dampens clock gene cycling amplitude

**Fig 2:**
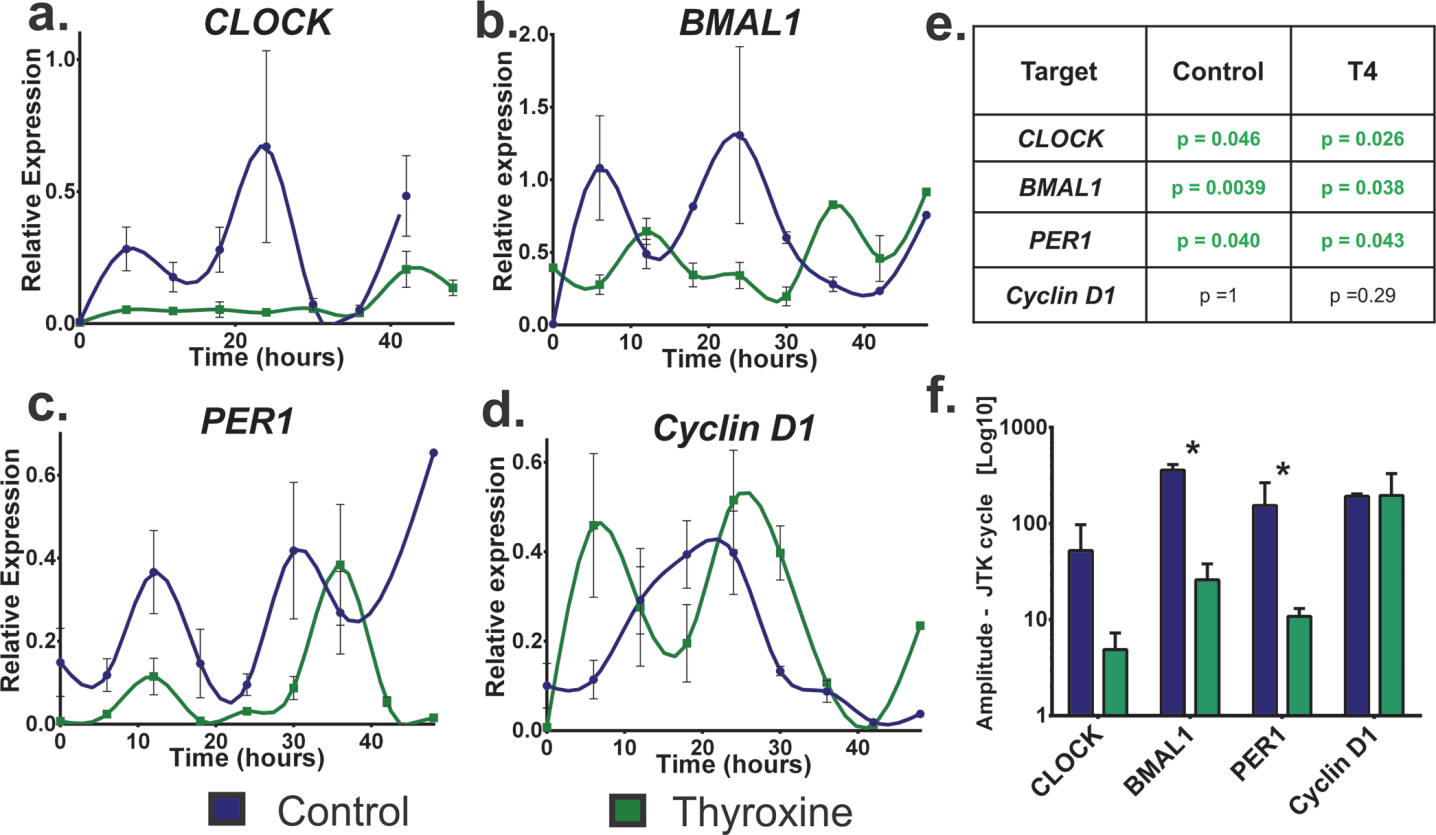
Thyroxine dampens clock gene expression over 48 hours. To assess the influence of T4 of on the circadian expression of *CLOCK*, *BMAL1*, *PER1* and *Cyclin D1*, HFs were synchronised, treated with T4 and sampled every 6 hours for 48 hours. (a, b, c & e) Quantitative-RT-PCR of clock transcripts showed that whilst both control and T4 treated HFs had rhythmic clock gene expression, which was supported by the confidence p values produced by the JTK cycle algorithm (e); it was reduced by T4 treatment. This was quantified by comparing the estimated amplitude measurement which demonstrated that the reduction in amplitude by T4 was significant for *BMAL1* and *PER1* (f). (d) Key cell cycle progression marker Cyclin D1 did not show a circadian expression pattern in the control group however, this appeared to be induced qualitatively by T4. (e) However this was not significant. (Mann-Whitney, * p < 0.05, mean (SD), HFs were pooled data from 4 donors).

To investigate whether the decrease in clock gene/protein expression was due to a loss of HF synchronisation and/or circadian rhythmicity, HF clock gene expression was assessed every 6 hours for 48 hours and the rhythmicity and amplitude was assessed using the JTK cycle algorithm (University of Missouri-St. Louis, MO, USA version 2.1) [47]. (**Fig 2A–2C**) These time course experiments demonstrated that over 48 hours HFs showed, both qualitatively and quantitatively, rhythmic expression of *BMAL1*, *CLOCK* and *PER1* in both control and T4 treated HFs (**Fig 2E**) [47]. However, while rhythmicity was maintained it was decreased by T4. To check for statistical significance JTK cycle was again utilised and the estimated amplitude measurement used to compare control and treated HFs (**Fig 2F**) [48]. Both *BMAL1* (p = 0.029) and *PER1* (p = 0.028) showed a significant decrease in amplitude when treated with T4 (**Fig 2F**). These data imply that whilst circadian rhythmicity was maintained, the clock gene transcript levels were lower, suggesting that T4 and may prolong anagen by reducing the overall amplitude of clock gene, specifically *BMAL1* and *PER1* expression in human HFs *in situ*.

### Thyroxine modulation of peripheral clock activity is via cyclin D1?

In order to probe potential mechanisms for how T4 modulation of clock activity impacts on HF biology, we next investigated the effects of T4 stimulation on cyclin D1, a key cell cycle-regulatory gene that is well-recognized and as a regulator of human hair matrix keratinocyte proliferation [49,50]. (**Fig 2D and 2E**) We did not observe a 24 hour rhythm in cyclin D1 in control HF expression however it did appear to oscillate over the time course assessed. This may be due to its expression changing through the cell-cycle. (**Fig 2D and 2E**).

### Long-term T4 treatment up-regulates clock gene mRNA and PER1 protein levels

**Fig 3:**
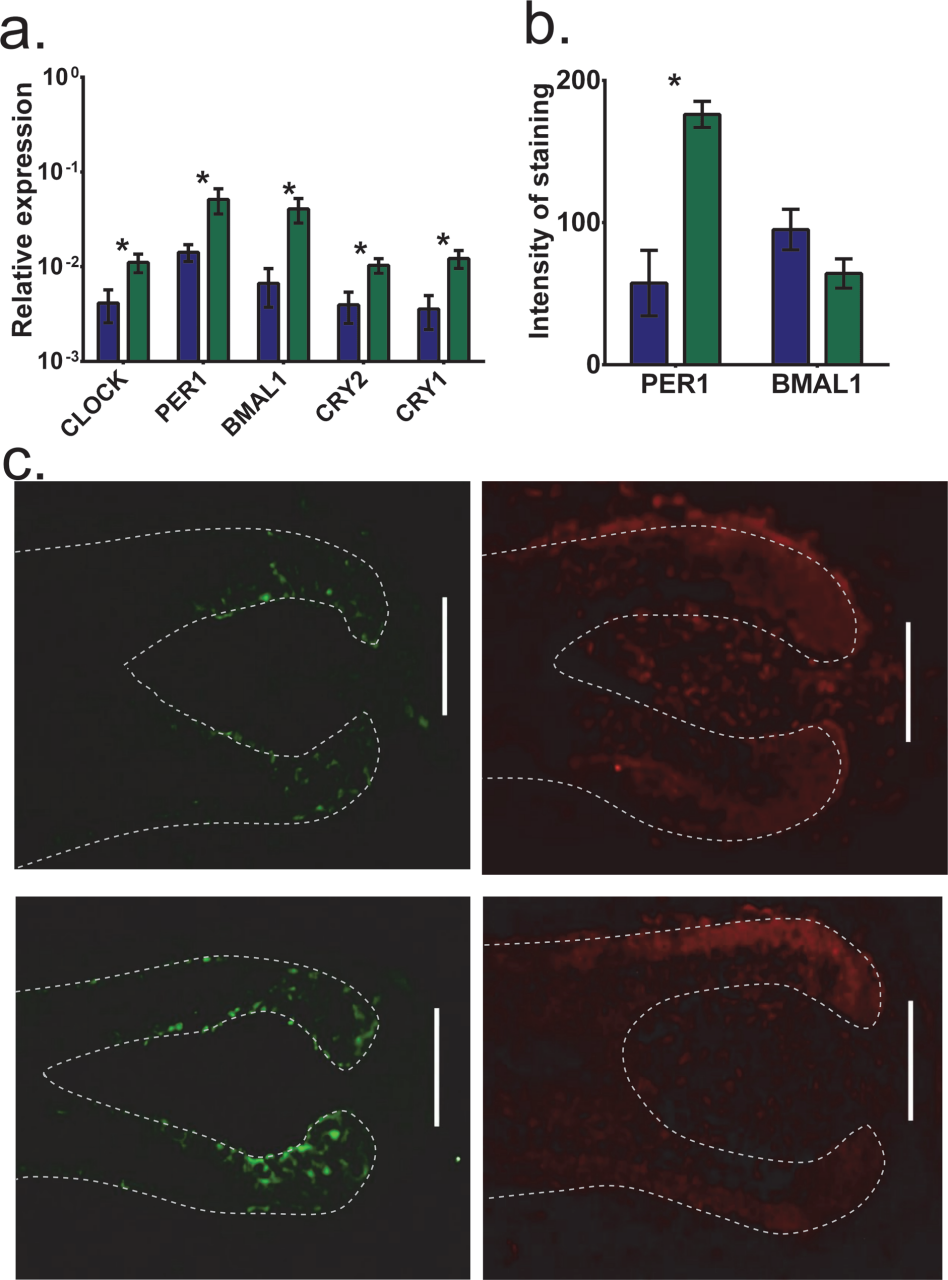
Clock transcript levels and PER1 protein levels are increased after 6 days treatment with T4. The effects of T4 on clock gene and protein expression were assessed on HFs treated for 6 days by immunofluorescence and qRT-PCR. (a) All core clock genes were significantly up-regulated at 6 days by T4 treatment. The Protein expression of PER1 was also up-regulated after 6 days (b & c) however, BMAL1 expression remained unchanged (b & D). (Mann-Whitney, * p < 0.05, mean (SD), HFs were pooled data from 3 patients). (scale bar = 50μm)

To assess how long-term administration of T4 affects intrafollicular clock activity, transcript and protein levels of core clock genes *BMAL1* and *PER1* was assessed after 6 days. This showed that, in contrast to HFs cultured for 6 and 24 hours, transcript levels of core clock genes (*CLOCK* [p = 0.030], *PER1* [p = 0.029], *BMAL1* [p = 0.013], *CRY1* [p = 0.010] and *CRY2* [p = 0.014]) were significantly up-regulated by T4, as assessed by qRT-PCR (**Fig 3A**).

Next, to evaluate whether this regulation occurred only at the level of transcription or translated also onto the protein level, human HF sections were immunostained for BMAL1 and PER1 after 6 days of HF organ culture in the presence of T4. Quantitative immunohistomorphometry showed that there was a significant increase in PER1 protein expression in the human HF (**Fig 3B and 3C**) after 6 day treatment with T4 (p = 0.017). In contrast, BMAL1 protein expression did not change in T4 treated HFs (**Fig 3B and 3D**). These findings suggest that selected intrafollicularly expressed clock genes are up-regulated by long-term T4 treatment on both the transcript and protein level.

### PER1 and BMAL1 transcript levels are increased in bulge stem cells

This observation raised the question; whether epithelial HF stem cells express clock genes *in situ* and whether any such expression is regulated by T4. Therefore, we asked whether the bulge region of human HFs expresses clock genes and whether this expression is regulated by T4.

**Fig 4:**
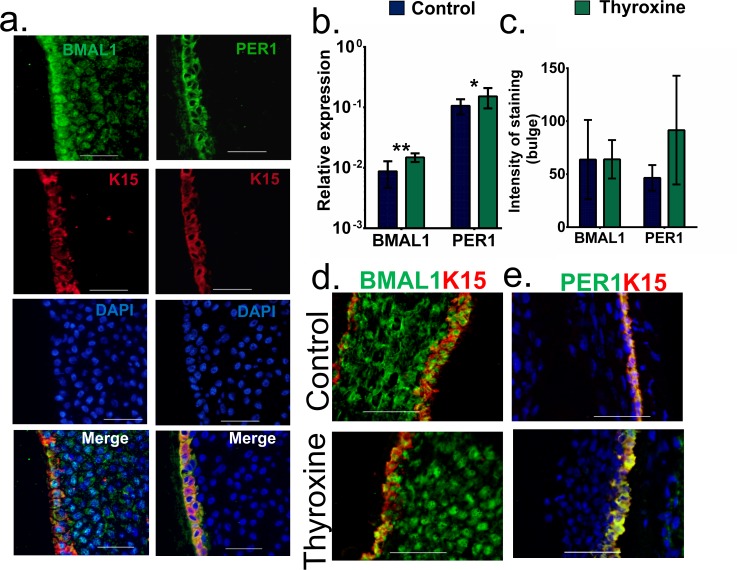
K15+ stem cells express BMAL1 and PER1 protein. (a) After establishing that K15 positive cells express BMAL1 and PER1 the role T4 on clock gene expression in the k15+ bulge stem cells was assessed by qRT-PCR and quantitative immunohistomorphometry. Transcript levels of BMAL1 and PER1 were upregulated after 6 days (b), (c-e) however, whilst PER1 showed a tendency to increase, neither BMAL1 or PER1 protein levels increased significantly. (scale bar = 50 μm, HFs were pooled from three donors, p < 0.05 *, ** p < 0.01, mean (SD)).

First, it was necessary to establish whether K15+ epithelial progenitor cells in the bulge/isthmus region of the HF epithelium cells express clock genes *in situ*. Using a dual stain for either PER1 and BMAL1 we were able to show that the stem cells do express both BMAL1 and PER1 (**Fig 4A**).

To analyse this, following culture with T4, human anagen VI HFs were first bisected and the upper (distal) part of the HF epithelium (well above the bulb), which contained all the bulge-associated HF stem cells, was analysed by qRT-PCR. This demonstrated that not only did the bulge stem cells express clock genes, but also that their expression was significantly increased following 6 day treatment with T4 (*BMAL1* (p = 0.001) *PER1* (p = 0.04) (**Fig 4B**). However, quantitative immunohistomorphometry of PER1 and BMAL1 protein demonstrated that T4 did not significantly change BMAL1 protein levels in K15+ cells *in situ* (**Fig 4D and 4E**). This may suggest that T4 regulates clock gene expression in human epithelial progenitor cells primarily at the level of transcription.

## Discussion

Here we present the first evidence that T4 is a modulator of peripheral clock activity in a model human (mini-)organ in the absence of central clock inputs. We show that T4 differentially modulates clock gene activity, with short-term stimulation significantly reducing intrafollicular transcript and protein levels of core clock members *BMAL1* and *PER1*, while circadian rhythmicity of intrafollicular gene expression is maintained. In contrast, long-term T4 stimulation up-regulates transcript and/or protein levels of all core clock genes (*BMAL1*, *PER1*, *CLOCK*, *CRY1*, *and CRY2)*. The effect of T4 on intrafollicular clock activity is likely to have a functional impact on HF biology, since qualitatively it appears to induce circadian rhythmicity of intrafollicular cyclin D1 expression (non-significant) and *BMAL1* and *PER1* are expressed in the bulge, the niche for HF epithelial stem cells.

That T4 reduces the gene and protein levels of BMAL1 and PER1 irrespective of the time of day, two core clock genes whose knock-down promotes hair growth, prolongs anagen and induces pigmentation [26,32] raises the possibility that the anagen-prolonging and pigmentation stimulatory effects of T4 in human scalp HFs [34] are, at least in part, mediated by reducing the intrafollicular activity of these clock genes mimicking the effects of clock knock-down. However, this hypothesis could not be definitively tested as it is not yet possible to *selectively* up-regulate BMAL1 and/or PER1 expression and activity in a human organ (e.g. by engineered overexpression).

This study documents that peripheral clock activity is hormonally regulated in the human HF. However, in order to convincingly support this claim we needed to demonstrate that any differences observed were not simply caused by normal diurnal changes in clock gene expression patterns. Therefore, intrafollicular clock activity was synchronised by dexamethasone treatment [51] and analysed using an algorithm designed to detect circadian expression patterns, JTK cycle [47]. This allowed us to conclusively demonstrate that the observed changes in HF clock gene and protein expression were genuinely caused by T4, rather than by constitutive circadian oscillations.

The regulation of the peripheral clock by T4 reported here is not surprising as T4 has long been associated with regulation of the molecular clock. For example, the thyroid gland influences clock circadian oscillations [24], and triiodothyronine (T3), which is intracellularly metabolised from T4 and is considered to represent the main biologically active TH [41]; is needed for development of the central circadian clock [52]. Moreover, thyroidectomy dampens *PER2* oscillatory activity in rats [42], while T4 shows distinct diurnal expression patterns, along with thyrotropin and triiodothyronine (T3) [53], and is essential for seasonal rhythms and mating season timing [40,41]. Despite this, our data is the first to demonstrate the direct role of T4 in modulating peripheral clock activity in human tissues *in situ*.

As cyclin D1 is a key player in cell-cycle progression, namely during the G2/M transition [54] and since the circadian clock is tightly coupled to cell-cycle progression [5,55,56] the effect of T4 treatment on cyclin D1 was investigated [35]. Although there was no significant difference in the average amplitude of expression, in the time course experiment, T4 treated HFs appeared to induce circadian rhythmicity in cyclin D1 expression. That T4 could be able to induce rhythmic expression of cyclin D1 suggests that the anagen-prolonging effect of T4 may be mediated by maintaining robust circadian expression of cyclin D1 over time. Although this was not statistically shown to be circadian, qualitative assessment suggests the expression may be circadian. There are a variety of explanations for the lack of significance, namely that the HF is made up of many different cell types which may each be influenced differently by T4 adding additional variation not only between patients but within the HF itself. This is supported by recent work that suggest the HF may have multiple clocks [26,32]. Secondly, that time points were taken every 6 hours. By reducing the sampling time, for example to 2 hours, would improve the data set and may show a significant results. However, due to the number of HFs required per time point it is difficult to do this experimentally. It would be desirable to sample more often and extend the time course beyond 48 hours conclusively determine whether cyclin D1 shows a circadian expression pattern when stimulated by T4.

If proven this observation would make biological sense as PER can inhibit cyclin D1 and therefore cell cycle progression [45,57]. The demonstrated reduction of PER by T4 may remove the regulatory effect of PER1 allowing the cell-cycle to occur quicker, leading to increased proliferation and therefore anagen prolongation observed. As a reduction in *Bmal1* increases proliferation and cyclin D1 expression in both murine cells and tumours, as well as decreasing apoptosis [58]. Furthermore, a reduction of *BMAL1* and *PER1* in humans is also associated with an increase in cyclin D1 expression and cell progression further supports this [50]. Previous work has suggested that cyclin D1 may also mediate the transition from bulge stem cells to their more differentiated, rapidly proliferating progeny (transient amplifying cells) in the suprabasal ORS of human HFs [49]. It should be noted that in the time course experiments there is often a shift in cycling peaking at different times of day, this is to be expected as thyroid hormones have been shown to be able to lengthen circadian periodicity [24].

As long-term T4 treatment prolongs anagen [34] (**S1 Fig**) while high levels of clock proteins promote catagen [26], we asked whether the reduction in clock gene and protein levels was maintained by long-term T4 stimulation. Contrary to expectations, this was not observed, since transcript levels of all the core clock genes investigated were significantly up-regulated. However, this effect only translated to the level of protein expression for PER1. This suggests that the activity of the molecular clock as a system needs to be up-regulated in order to induce catagen, while up-regulation of just one of its protein components is insufficient. Alternatively, the increase in clock transcript levels could mainly result from events in selected intrafollicular cell subpopulations outside of the hair matrix. Interestingly, long-term T4 treatment also increased clock gene expression in bulge epithelial stem cells (**Fig 4**). Moreover, long-term TH receptor stimulation may induce complex gene regulation cascades that impact on intrafollicular clock gene expression and reverse the effects of a short-term pulse of T4 treatment. One potential mechanism is via T4 conversion to T3 which in turn leads to production NCOR1. NCOR1 is believed to associate with the accessory clock loop involving REV-ERBα with ultimately represses BMAL1, CLOCK and therefore PER1 production [59].

There are many additional factors to consider in relation to the core clock including histone modifications [60,61], post translational modulations and the potential for novel clock genes such as *CHRONO* [61]. Other factors include alternate splicing such as the U2-auxillary-factor 26 (U2AF26) variant which leads to destabilisation of PER1 [62] and microRNAs such as miR192/194 which regulates PER [63]. It could be one or a variety of these factors that link thyroid hormones with HF molecular clock activity or via direct modulation of TH transcription. In regards to the latter however, whilst there is a sequence matching reported thyroid response element sequences (T(A/G)AGGTCA) [64] in the sequence for *BMAL1* (From ENSEMBL), this does not occur in the promoter region [65]. This render it unlikely that the transcriptional regulation of these genes via thyroxine operates in the classical TRE-mediated manner. An alternative possibility is that clock genes regulate Deiodinase 2 (DIO2) enzyme which converts T4 to the more active triiodothyroinine (T3) in a manner similar to that in the hypothalamus [66]. This would need to be examined I future experiments.

The evidence provided here that T4 modulates not only the central, but also the peripheral clock, namely in human HFs, has important clinical implications. Following up the current study on the clinical level, it will be interesting to examine, next, whether and how the expression of PER1 and BMAL1 mRNA and protein change in the scalp skin of patients with thyroid dysfunction, even though confounding complex (neuro-) endocrinological abnormalities must be taken into account in such patients that could have altered peripheral clock expression/activity indirectly.

Our observations suggest that patients suffering from thyroid dysfunction are likely to also show a disordered peripheral clock and that this may contribute to the overall pathology associated with abnormally low or high serum TH levels. Furthermore, our study raises the intriguing possibility that, in diseases associated with clock dysfunction, such as hypertension and type 2 diabetes [9–11], short-term, pulsatile treatment with T4 might permit one to modulate circadian activity in diseased peripheral tissues in a therapeutically beneficial manner, therefore providing a novel strategy for differential endocrine “peripheral clock therapy”.

## Materials and Methods

### Human hair follicle collection and culture

Discarded human HFs from hair transplant surgery were obtained with full written consent adhering to the ‘declaration of Helsinki principles’. Human tissue was stored in a registered biobank following human tissue act guidelines and stored with ethical and institutional approval from the University of Manchester.

### Human organ culture

Human scalp HFs were dissected on the day of surgery from surrounding tissue and cultured at 37°C with 5% CO_2_ in a minimal media of William’s E media (Gibco, Life technologies) supplemented with 2mM of L-glutamine (Gibco), 10ng/ml hydrocortisone (Sigma-Aldrich), 10μg/ml insulin (Sigma-Aldrich) and 1% penicillin/streptomycin mix (Gibco) [67]. On the following day HFs were treated with 100nM of dexamethasone for 30 minutes to rest HF clock activity at 8:30am so all HFs had synchronised activity. At 9am HFs were cultured in minimal media (William’s E media with clinically physiologically relevant levels of T4 (100nM) [35] (Sigma-Aldrich, Dorset, UK) with a parallel control. HFs were either snap frozen in OCT or RNAlater (Ambion, Connecticut, USA). For 6 day culture HFs were taken at 9am and 3pm to exclude that any differences in protein level were caused by any diurnal expression changes.

### Time course experiments

For time course experiments HF clock activity was synchronised and HFs for 30 minutes with dexamethasone. Following synchronisation HFs were cultured with 100nM T4. HFs were removed from culture immediately after synchronisation (0 hours) and then subsequently sampled every 6 hours for 48 hours. Five HFs were taken from control and treated at each time point which were pooled for RNA extraction. Four time course cultures were completed in total, two donors for 48 hours and an additional two for 24 hours. For the immunofluorescence experiments, 6 hair follicles were embedded in OCT at each time point (0–24 hours) per donor (3 donors used in total for protein data).

### RNA extraction and qRT-PCR

HF RNA was extracted using an RNeasy micro kit (Qiagen, Manchester, UK). Complimentary DNA was synthesised from this using a Tetro cDNA synthesis kit (Bioline, London, UK) and used for qRT-PCR at 10ng/μl per well. Quantitative-PCR was performed using the StepOne real time PCR system (Applied Biosystems, Warrington, UK) using Taqman (Applied Biosystems) fast Advance master mix and probes listed in S1 Table. Results were performed in triplicate for each experiment. For experiments looking at the bulge clock gene expression HFs were bisected with a scalpel above the HF bulb separating the proliferating bulb and the upper ORS containing the bulge region. RNA was extracted from each region separately.

### Immunofluorescence

Human HF cryosections (5μm thick) were fixed at -20°C in acetone (PER1, rabbit-anti human PER1 (Santa Cruz)) stain or methanol (BMAL1, rabbit-anti human (genetex)). HFs were washed in PBS and pre-incubated with 10% normal goat serum. For dual stains with K15, the protocol for either PER1 or BMAL1 was followed with the addition of mouse- anti K15 (Abcam, Cambridge, UK). Clock proteins were stained green using goat- anti rabbit Alexafluor 488 (Life technologies, Paisley, UK) secondary antibody. K15 was visualised in red using goat-anti-mouse Alexafluore 594. HF sections were subsequently imaged using a Keyence Biozero fluorescence microscope (Osaka, Japan).

### Immunohistomorphometry

Using the microscope images, sections were analysed using image J. Images were converted to 8-bit to reduce the background. Three background measurements were taken and subtracted from fluorescence readings (corrected fluorescence). To measure fluorescence two rectangles of set size were used encompassing the positive signal, one on the right of the dermal papilla and one on the right. This methodology was modified from previous studies [26,32,68]. Measurements were taken from three images for each HF from multiple HFs and donors.

### Statistical analysis

All statistical analysis was performed using Graphpad prism 6. Data was first assessed for normality using a D’Agostino-Pearson test. For normally distributed data, to test for significance, a two-tailed student’s Ttest was used. Where the data did not follow a normal distribution a Mann-Whitney test was used. Data was corrected for multiple comparisons using the Holm-Sidak equation. Data was considered statistically significant if the P value was less than 0.05.

The software package R (version 3.1.2) [69] was used to assess quantitatively whether transcripts oscillated over using the JTK cycle algorithm (version 2.1) [47]. To assess whether there was a difference in amplitude between control and T4 treated HFs the estimated amplitude (AMP) measurement calculated for each donor for each gene was compared using a Mann-Whitney test.

## Supporting Information

S1 Fig Confirmation of the role of thyroxine (T4) on hair follicle (HF) physiology. Thyroxine has been shown to promote melanin production in HFs and prolong anagen. To confirm this HFs were cultured in the presence or absence of T4 and the percentage remaining in anagen (a.) was assessed by morphological criteria and melanin content was assessed by Masson-Fontana. Results demonstrated that in the T4 treated group, a significantly higher percentage of HFs remained in anagen (a.) and had a higher melanin content (b.) confirming previous results (mean (SD), * p<0.05, ** p<0.001, Student’s Ttest).
(PDF)

S1 Table A list of Taqman advance probes used for qRT-PCR. (PDF)
