# Supplementary material for: Thyroxine Differentially Modulates the Peripheral Clock: Lessons from the Human Hair Follicle
Source: PLoS One. 2015 Mar 30;10(3):e0121878. doi: 10.1371/journal.pone.0121878 (PMC4379003; doi:10.1371/journal.pone.0121878)
Supplement: S1 Fig — Thyroxine has been shown to promote melanin production in HFs and prolong anagen. To confirm this HFs were cultured in the presence or absence of T4 and the percentage remaining in anagen (a.) was assessed by morphological criteria and melanin content was assessed by Masson-Fontana. Results demonstrated that in the T4 treated group, a significantly higher percentage of HFs remained in anagen (a.) and had a higher melanin content (b.) confirming previous results (mean (SD), * p<0.05, ** p<0.001, Student’s Ttest). (PDF) [file pone.0121878.s001.pdf]

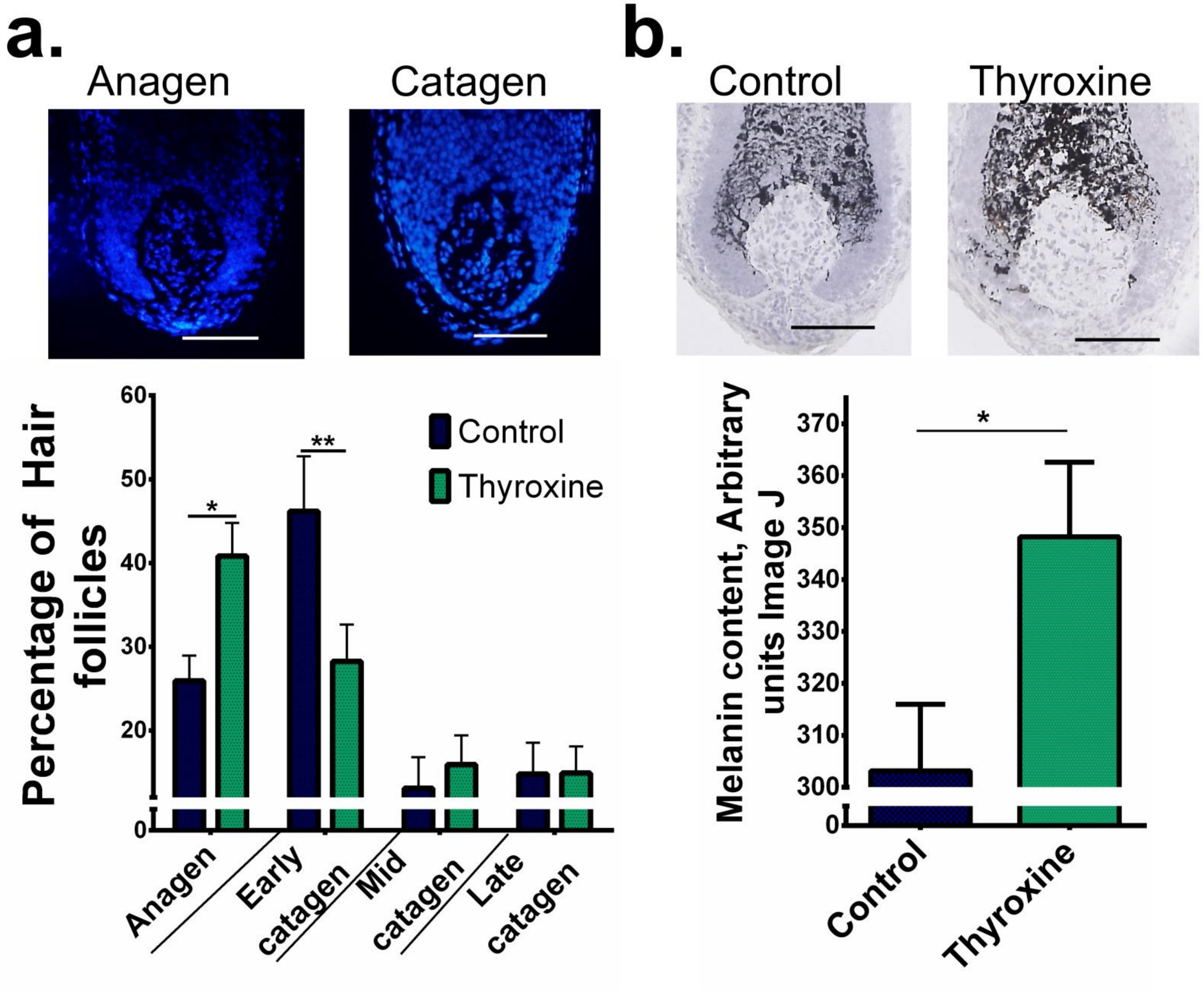

Supplementary Fig. S1: Confirmation of the role of thyroxine (T4) on hair follicle (HF) physiology. Thyroxine has been shown to promote melanin production in HFs and prolong anagen. To confirm this HFs were cultured and the percentage remaining in anagen(a.) was assessed by morphological criteria and melanin content was assessed by Masson-Fontana. Results demonstrated that in the treated group a significantly higher percentage of HFs remained in anagen (a.) and had a higher melanin content (b.) confirming previous results (error bars = ±SEM, * $p<0.05$, ** $p<0.001$, Student's Ttest).
