# Supplementary material for: Thyroxine Differentially Modulates the Peripheral Clock: Lessons from the Human Hair Follicle
Source: PLoS One. 2015 Mar 30;10(3):e0121878. doi: 10.1371/journal.pone.0121878 (PMC4379003; doi:10.1371/journal.pone.0121878)
Supplement: S1 Table — (PDF) [file pone.0121878.s002.pdf]

Gene	Taqman® assay ID	Full gene name
PPIA	**Hs99999904_m1**	Peptidylprolyl isomerase A
GAPDH	**Hs02758991_g1**	Glyceraldehyde 3-phosphate dehydrogenase
CLOCK	**Hs00231857_m1**	Circadian-locomotor output cycle kaput (clock homolog (mouse)
BMAL1	**Hs00154147_m1**	aryl hydrocarbon receptor nuclear translocator-like
PER1	**Hs00242988_m1**	Period homolog 1 (Drosophila)
CRY1	**Hs01597804_m1**	Cryptochrome-1
CRY2	**Hs00901396_m1**	Cryptochrome-2
K15	**Hs00267035_m1**	Cytokeratin 15
CCND1	**HS00765553_m1**	Cyclin D1

Supplementary Table S1: A list of Taqman® advance probes used for qRT-PCR.
